# The mechanoresponse of bone is closely related to the osteocyte lacunocanalicular network architecture

**DOI:** 10.1073/pnas.2011504117

**Published:** 2020-12-07

**Authors:** Alexander Franciscus van Tol, Victoria Schemenz, Wolfgang Wagermaier, Andreas Roschger, Hajar Razi, Isabela Vitienes, Peter Fratzl, Bettina M. Willie, Richard Weinkamer

**Affiliations:** ^a^Department of Biomaterials, Max Planck Institute of Colloids and Interfaces, 14476 Potsdam, Germany;; ^b^Berlin-Brandenburg School for Regenerative Therapies, Charité–Universitätsmedizin Berlin, 13353 Berlin, Germany;; ^c^Department of Chemistry and Physics of Materials, Paris Lodron University of Salzburg, A-5020 Salzburg, Austria;; ^d^Research Centre, Shriners Hospital for Children-Canada, Montreal, H4A 0A9, Canada;; ^e^Department of Pediatric Surgery, McGill University, Montreal, H3A 0G4, Canada

**Keywords:** bone adaptation, mechanobiology, fluid flow, lacunocanalicular network, in vivo µCT

## Abstract

Organisms rely on mechanosensing mechanisms to adapt to changes in their mechanical environment. Fluid-filled network structures not only ensure efficient transport but can also be employed for mechanosensation. The lacunocanalicular network (LCN) is a fluid-filled network structure, which pervades our bones and accommodates a cell network of osteocytes. For the mechanism of mechanosensation, it was hypothesized that load-induced fluid flow results in forces that can be sensed by the cells. We use a controlled in vivo loading experiment on murine tibiae to test this hypothesis, whereby the mechanoresponse was quantified experimentally by in vivo micro-computed tomography (µCT) in terms of formed and resorbed bone volume. By imaging the LCN using confocal microscopy in bone volumes covering the entire cross-section of mouse tibiae and by calculating the fluid flow in the three-dimensional (3D) network, we could perform a direct comparison between predictions based on fluid flow velocity and the experimentally measured mechanoresponse. While local strain distributions estimated by finite-element analysis incorrectly predicts preferred bone formation on the periosteal surface, we demonstrate that additional consideration of the LCN architecture not only corrects this erroneous bias in the prediction but also explains observed differences in the mechanosensitivity between the three investigated mice. We also identified the presence of vascular channels as an important mechanism to locally reduce fluid flow. Flow velocities increased for a convergent network structure where all of the flow is channeled into fewer canaliculi. We conclude that, besides mechanical loading, LCN architecture should be considered as a key determinant of bone adaptation.

## Significance

The explanation of how bone senses and adapts to mechanical stimulation still relies on hypotheses. The fluid flow hypothesis claims that a load-induced fluid flow through the lacunocanalicular network can be sensed by osteocytes, which reside within the network structure. We show that considering the network architecture results in a better prediction of bone remodeling than mechanical strain alone. This was done by calculating the fluid flow through the lacunocanalicular network in bone volumes covering the complete cross-sections of mouse tibiae, which underwent controlled in vivo loading. The established relationship between mechanosensitivity and network architecture in individual animals implies possibilities for patient-specific therapies. A new connectomics approach to analyze lacunocanalicular network properties is necessary to understand skeletal mechanobiology.

Fluid-filled network structures are used in many organisms for transport and signaling by making use of their excellent pervasion of tissues, while requiring only a limited volume. A variety of tissues and organs use fluid flow in networks to sense the mechanical environment, thereby contributing to their morphogenesis, active maintenance, and adaptation to changing demands (1). Important examples include the formation of the circulation and nervous system (2, 3), both the rapid and long-term adaptation of the lungs (4), and the adaptation of bone to mechanical loads (5–7). Important distinguishing characteristics between networks are the mechanical flexibility of the walls of their channels and their network architecture. The cardiovascular circulation is an example of a tree-like network with channels repeatedly branching, which induces constraints on the diameters of the vessels for an efficient flow distribution (8). The circulation network is not static but can adapt its network architecture, for example, by maintaining or disconnecting arterial side branches based on locally sensed blood flow velocities (2). The multifunctionality of fluid-filled networks is interesting from an evolutionary viewpoint, since most likely the functions were not established at the same point in time (9). Their potential has also been explored in man-made so-called vascular materials, where the fluid-filled network should impart self-healing properties to the material (10, 11).

In bone, fluid flow occurs in the lacunocanalicular network (LCN), a porous network of micrometer-sized lacunae connected by roughly 300-nm-wide canals, called canaliculi (12, 13). The lacunae accommodate the cell bodies of osteocytes, while their cell processes run within the canaliculi. The pericellular space between the cell membrane and the mineralized bone tissue is filled by a proteoglycan-rich matrix (glycocalyx) and by an interstitial fluid. Deformations occurring through the bone matrix are too small to be directly sensed by bone cells since bone has a high stiffness, compared to cells, of roughly 10 GPa. Therefore, a strain amplification mechanism involving the interstitial fluid was proposed (12). The fluid flow hypothesis states that external loading deforms the bone including the porous network. The deformation of the porous network induces a flow of the fluid through the LCN toward or away from free bone surfaces (14). With typical loading frequencies (such as walking) being in the 1-Hz range, the load-induced fluid flow should not be imagined as a substantial fluid transport, but rather as fluid oscillating back and forth. The oscillating fluid flow results in drag forces on osteocytes that are sufficiently strong to trigger a mechanoresponse (15). Important findings in favor of the fluid flow hypothesis are as follows: 1) osteocytes are the most mechanosensitive cells in bone (16, 17), in particular to pulsatile fluid flow (18) with their cell processes sensing shear forces (19); the primary cilium could act as an additional mechanosensor for fluid flow (20); 2) dynamic loads induce fluid displacement through the LCN in vivo (21); 3) theoretical models predict load-induced fluid flow velocities of magnitudes, which osteocytes respond to in vitro (22); 4) specific detection mechanisms have been proposed, which stress the importance of the glycocalyx with its tethering fibers for the transmission of forces to cell processes (23). In addition, the cell process is attached by integrins to canalicular projections, which are infrequent, discrete locations along the canalicular wall (19, 24, 25).

While important progress was made in characterizing molecular and cellular aspects of bone’s mechanotransduction, the connection between mechanical stimulation of the bone and its mechanoresponse in the form of bone formation and resorption is still not satisfactorily established. The challenge is to define a mechanical stimulus, which can be spatially correlated to locations of bone formation and resorption. The most successful attempts until now have been based on strain-related mechanical stimuli (26, 27). In a recent mouse study, strain energy density and fluid flow velocity were used as predictor for the mechanoresponse in the tibia (28). The spatial fluid velocity pattern was calculated based on a continuum model without considering the LCN architecture and, therefore, reflects only the strain state. The calculated fluid flow could not explain the higher mechanoresponse observed at the endosteal surface compared to the periosteal surface (28–30). It is obvious that mechanical strain and load-induced fluid flow through a network-like structure are very different physical quantities. Since the fluid flow through the LCN is challenging to assess experimentally, computational models are frequently employed to calculate flow velocities through the canaliculi. However, the network architectures of these models were either restricted to small parts of the network (like one lacuna and emerging canaliculi) (31) or unrealistically regular (32–34). Recent advances in imaging technology allow one to image and analyze the three-dimensional (3D) architecture of the LCN in much larger bone volumes. It was shown that the LCN architecture is spatially very heterogeneous (35–37) and changes with age (38–40). Combining such 3D imaging of the LCN with fluid flow calculations predicted substantial differences in the mechanoresponsiveness between different osteon types in human cortical bone (41).

The aim of this study is to test the fluid flow hypothesis by taking into account the architecture of the LCN and to predict where bone is formed or resorbed after mechanical stimulation. In three mice, the response to a controlled mechanical loading was quantified in terms of newly formed and resorbed bone on both the inner endocortical and the outer periosteal surfaces of the tibiae using time-lapse in vivo micro-computed tomography (µCT) following the protocol of Birkhold et al. (42). In the tibiae of the same mice, the LCN was imaged in bone volumes covering whole cross-sections of the tibiae. Circuit theory was then used to calculate the load-induced fluid flow through the LCN. As a result, this integration of mathematical modeling with experimental techniques allows us to perform a direct spatial correlation between predicted mechanoresponse based on fluid flow patterns in the actual LCN architecture and the measured mechanoresponse.

The detailed strategy to achieve our aim is based on the combination of six different experimental and computational techniques: 1) in vivo axial compressive loading of the mouse tibia to provide a well-defined anabolic loading regime (43), while 2) time-lapse in vivo µCT was used to monitor bone (re)modeling events over 15 d (42). 3) Local strain during the loading experiments was calculated using finite-element (FE) modeling (44). 4) The LCN was imaged in 3D using confocal microscopy after rhodamine staining. 5) A conversion of image data into a mathematical network was performed using a custom software (36). 6) Circuit theory was applied to calculate the load-induced fluid flow in each of the millions of imaged canaliculi (45), and a mechanical stimulus was inferred (41), which is used as a predictor of the mechanoresponse of the tibia.

## Results

### Structural Heterogeneity of the Mouse LCN.

**Fig. 1.**
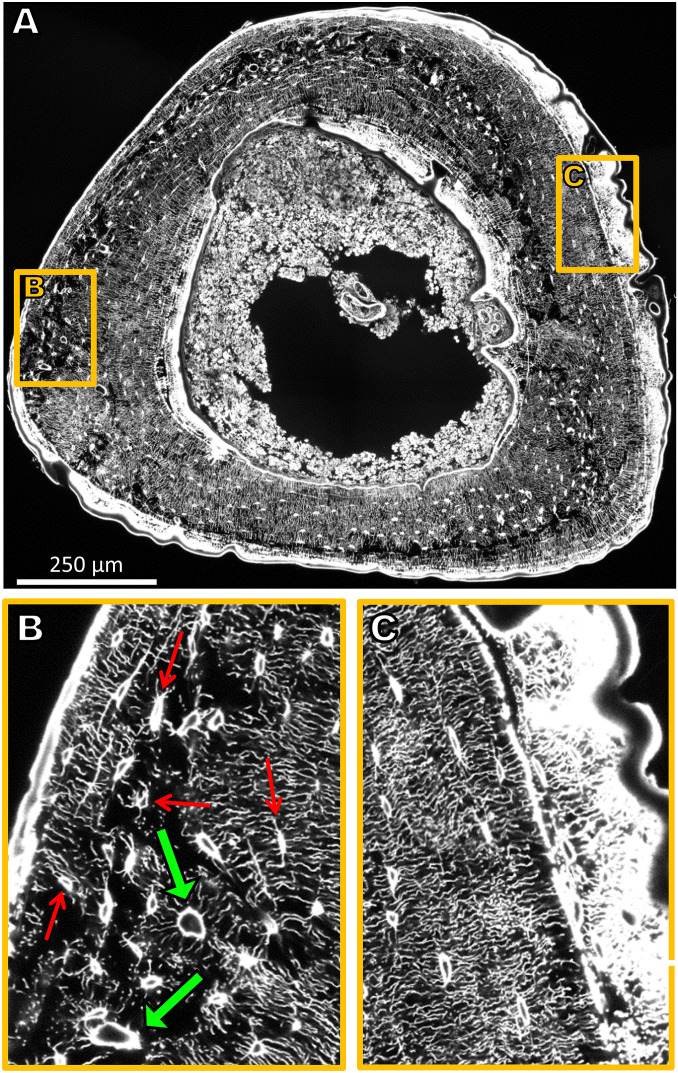
(*A*) Sixteen confocal laser-scanning microscopy (CLSM) image stacks stitched together amounting to a volume of 1,000 × 1,200 × 50 µm^3^ and covering the complete cross-section of the tibia. Due to the rhodamine staining, osteocyte lacunae and canaliculi are clearly visible. For reasons of presentation, a single 2D section of the 3D image is shown. (*B*) Enlargement of a region close to the periosteal surface with a loose network of low connectivity comprising vascular channels (green arrows). Some lacunae are marked with thin red arrows. (*C*) Enlargement of a region close to the periosteal surface with a dense, ordered, and well-connected LCN architecture. Newly formed bone as a response to mechanical stimulation (to the *Right*) is highly stained and, therefore, appears bright white.

**Fig. 2.**
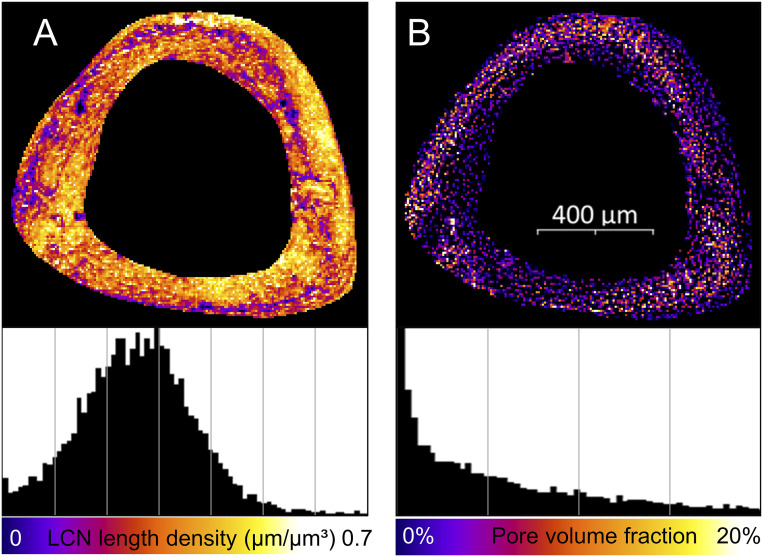
Structural heterogeneity of the lacunocanalicular network (LCN) within the tibial cross-section averaged over the imaging depth of 50 µm. (*A*) Map of the canalicular density (Ca.Dn, total length of canaliculi per unit volume). (*B*) Map of the pore density (i.e., volume of both lacunae and vascular canals per unit volume). Below, frequency distributions are shown for both quantities with *x*-axis ticks as lines.

The tibiae of three 26-wk-old female C57BL/J6 mice underwent 2 wk of controlled loading, and the LCN within these tibiae was imaged afterward. The 3D structure obtained by confocal imaging of the LCN in whole cross-sections of the tibia (Fig. 1*A*) revealed a heterogeneity of the LCN with regions of looser (Fig. 1*B*) and denser network (Fig. 1*C*) (for videos of the full image stacks, see clsm_mouse1/2/3.avi in ref. 46). A quantitative analysis of the network density in terms of canalicular density, Ca.Dn, (i.e., total length of canaliculi per unit volume) resulted in an average value of 0.27 µm/µm^3^ (Table 1). The frequency histogram (Fig. 2 *A*, *Bottom*) shows a broad bell-shaped distribution with a SD of 0.12 µm/µm^3^. A map of the spatial distribution of Ca.Dn (Fig. 2*A*) reveals that regions of low network density can be found in a band-like structure, which runs eccentrically in the cortex. Regions with a roughly 10-fold difference in network density can be found adjacent to each other. Evaluation of the pore volume fraction (i.e., contribution of lacunae and vascular channels to the porosity) (Fig. 2*B*) demonstrate that regions of high porosity spatially correlate with low network density regions (Fig. 2 *A* and *B*).

**Table 1.** Values of structural parameters and prediction quality

	Mouse 1	Mouse 2	Mouse 3	Averaged
Canalicular density, Ca.Dn, µm/µm^3^	0.27 ± 0.12	0.27 ± 0.11	0.26 ± 0.11	0.27 ± 0.12
Pore volume fraction, µm^3^/µm^3^	2.6 ± 3.8	2.0 ± 3.0	1.8 ± 2.6	2.1 ± 3.2
RMSE values of predictors, µm				
Fluid flow velocity: endocortical	21.8	13.7	12.8	11.5
Strain: endocortical	24.3	10.3	13.0	13.2
Fluid flow velocity: periosteal	16.7	9.0	15.0	10.0
Strain: periosteal	18.5	23.0	22.0	18.5

Values for the canalicular density and the pore volume fraction for all three mice (mean density ± SD of all 7.4 × 7.4 × 7.4-µm^3^ subvolumes) and values averaged over all three mice. Root-mean-square error (RMSE) assesses the prediction quality between the experimental mechanoresponse (Fig. 5*B*) and the prediction based on strain only (Fig. 5*A*) and fluid flow (Fig. 5*C*). The RMSE was calculated as RMSE $=\sqrt{\left(\sum_{i=1}^{N}{\left(\mathrm{predicte}d_{i}-\mathrm{measure}d_{i}\right)}^{2}\right)/N}$ with *N* = 180, and is given in micrometers. For the average, the prediction from strain/fluid flow was first averaged over all three mice, and then the RMSE was calculated.

### Bone Formation and Resorption as Response to Mechanical Loading.

**Fig. 3.**
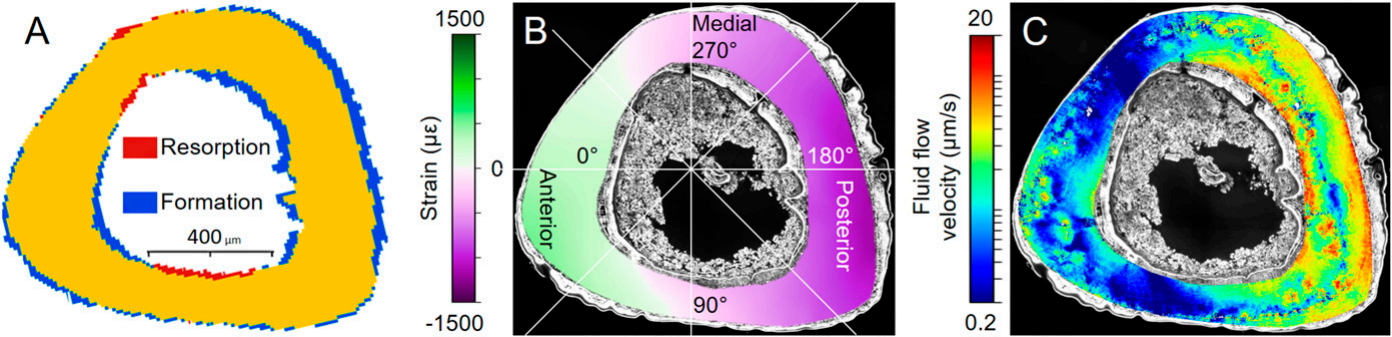
(*A*) Outcome of the in vivo µCT experiment showing where in the diaphyseal region of the tibia, bone was formed or resorbed in response to mechanical loading (blue denotes newly formed bone, red resorbed bone, and yellow quiescent bone; 2D cross-section of an imaged 3D volume). (*B*) Spatial distribution of the peak strains induced by the in vivo loading experiment calculated using FE modeling. Green colors correspond to tensile, and violet to compressive strains. The figure also introduces the angular coordinate system used to indicate locations at the endocortical and periosteal surfaces. The anterior direction is at 15°, and angles increase counterclockwise. (*C*) Pattern of fluid flow velocities through the LCN. Based on the loading conditions from *B* and the 3D network architecture of Fig. 1*A*, the fluid flow velocity is calculated in each canaliculus using circuit theory. The fluid flow velocity information of all of the canaliculi was rendered in a 3D image stack. For reasons of presentation, this 3D image is averaged over the imaging depth to obtain the shown flow pattern. Results shown are for mouse 1 (Fig. 5).

The registration of two in vivo µCT images (time lapse between measurements 15 d) provided information about where and how much bone was formed or resorbed on the endocortical and periosteal surfaces. Most bone formation is found at posterior sites (Fig. 3*A*, blue), much less in the anterior direction. Hardly any new bone is formed along the medial and lateral axis, while these are the locations where resorption was observed (Fig. 3*A*, red).

### Strain Distribution and Load-Induced Fluid Flow Pattern through LCN.

Based on a high-resolution ex vivo µCT scan and the experimental in vivo loading conditions, a FE model was used to calculate the bulk strain distributions of the whole tibia (Fig. 3*B*) (44). Since the tibia undergoes bending, the anterior region is under tension, while the posterior region is compressed. The highest strains are found close to the outer, periosteal surface with compressive strains larger than tensile strains (Fig. 3*B*). We combined this information about local strain rates with the imaged 3D network architecture to calculate the fluid flow velocity through each individual canaliculus employing circuit theory (see *Materials and Methods* for model details). The resulting fluid flow pattern has an interesting “hybrid” character (Fig. 3*C*). The pattern clearly reflects features of the strain distribution such as the low fluid flow around the mechanically neutral medial–lateral direction. However, some important features cannot be explained by strains and, therefore, have to be attributed to the LCN architecture: Examples are the high fluid flow velocities close to the endocortical surface at the posterior side or regions of low fluid flow at the anterior side of the tibia (Fig. 3*C*). Regions with low network density are spatially associated with low flow velocities.

### Predictors for Bone (Re)Modeling: Fluid Flow through LCN Compared to Strain.

**Fig. 4.**
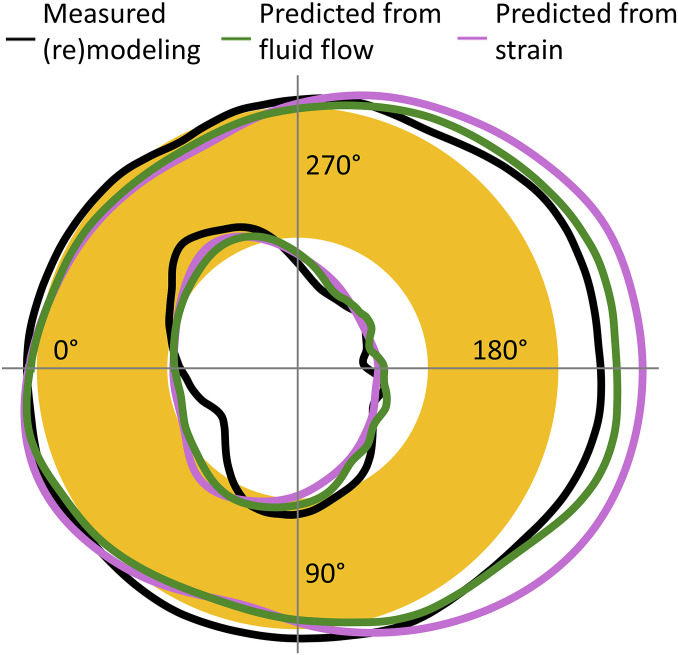
Result of in vivo µCT measurements in terms of bone formation and resorption after 2 wk of controlled loading of the tibia. The tibial cross-section is represented schematically as a circular annulus (yellow). The black line denotes the amount of resorbed bone (line entering yellow cortex) and formed bone (depiction not to scale). The pink line denotes the prediction of the mechanoresponse based on strain only; the green line is the prediction based on load-induced fluid flow, which considers not only the loading condition but also the architecture of the lacunocanalicular network (LCN). Strain rate and fluid flow velocity were integrated over regions close to the surface (*Materials and Methods*) to obtain a single value.

The result of the in vivo µCT experiments measuring the amount of bone formed or resorbed averaged over the three investigated mice is shown in Fig. 4 (black line). The schematic represents a transverse section through the midshaft of the mouse tibia. The amount of formed bone and resorbed bone (Fig. 4, black line entering the yellow ring) is not depicted to scale for reasons of clarity. The measured mechanoresponse is compared with the predictions from strain alone (pink line) and from load-induced fluid flow (green line). At the periosteal surface, the prediction from strain is overestimating the mechanoresponse at the posterior side. Quantification of the difference between prediction and measurement as a root-mean-square error (RMSE) gives a value of 18.5 µm for using strain as predictor and 10.0 µm predicted by fluid flow. At the endocortical surface, the error is only slightly improved for fluid flow as predictor and a substantial part of the error can be attributed to a poor prediction of resorption (RMSE = 13.2 µm for strain; 11.5 µm for fluid flow) (see Table 1 for all values).

**Fig. 5.**
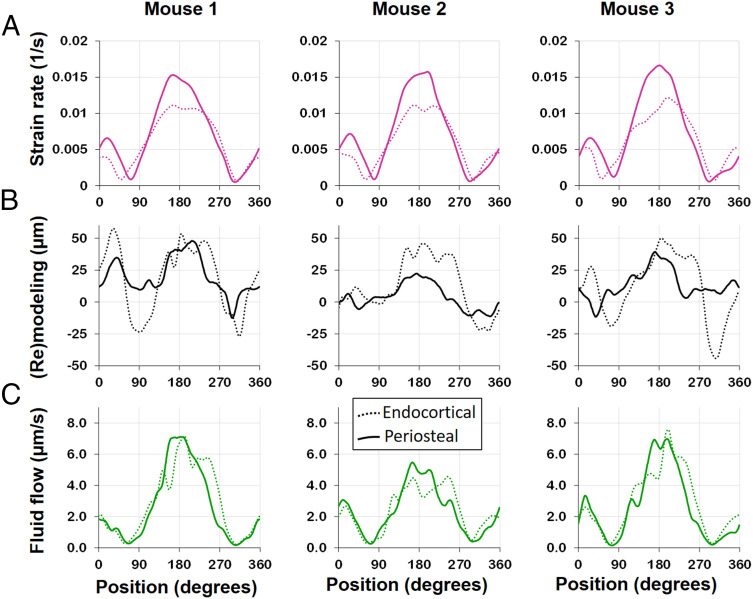
Evaluation of absolute surface strain rate (*A*), (re)modeling thickness (*B*), defined as new bone thickness minus resorption cavity depth, and surface fluid flow velocity (*C*) for all three investigated mice (see Fig. 3*B* for definition of angles). Strain rate and fluid flow velocity were integrated over regions close to the surface (*Materials and Methods*) to obtain a single value. (*A*) Strain rates at the endocortical surface (dotted line) are lower compared to the periosteal surface (solid line), and the spatial distribution and peak values are very similar between mice. (*B*) The mechanoresponse shows individual differences in bone (re)modeling, with mouse 2 showing less (re)modeling compared to the two other mice. (*C*) Also surface fluid velocity was found to be lower in mouse 2, while for all animals the flow velocities show similar distributions on the endocortical and periosteal surfaces.

Since the LCN exhibits architectural differences for the individual animals, the prediction quality has to be assessed on the basis of the specific animals. Fig. 5 summarizes the outcome of the in vivo µCT experiment (Fig. 5*B*) and the predictions of the mechanoresponse for strain (Fig. 5*A*) and fluid flow (Fig. 5*C*) for all three investigated mice. The angle on the *x* axis specifies the position at the surface (Fig. 3*B*). The two lines in all plots refer to the endocortical and the periosteal surface, respectively. In Fig. 5*B*, the value on the *y* axis denotes the thickness of the formed/resorbed bone, where a binning angle of 2° was used followed by 30° triangular moving average. This thickness is defined as the total formed/resorbed volume divided by the surface area and is positive/negative for predominant formation/resorption. The sine wave-like curves show that formation is strongest around 190° (posterior direction) with a second smaller maximum at about 10° (anterior direction) and small minima (corresponding to resorption) in between (i.e., at bone’s neutral axes). Two observations can be made: 1) The mechanoresponse is similar on both surfaces with a trend to higher values at the endocortical surface; 2) the mechanoresponse in the three mice is substantially different, with mouse 1 showing the strongest response, followed by mouse 3 and mouse 2. Fig. 5*C* shows the evaluation of the fluid flow velocity through the LCN close to the surface (see *Materials and Methods* for details), plotted similarly as the (re)modeling response. Also, the average flow velocities show the rough sine wave curves with maxima and minima at positions similar to the (re)modeling response with the strongest maximum again at roughly 190°. The flow velocities are similar for both surfaces, but different for different animals: Mouse 1 displays the largest values for the fluid flow velocities, while mouse 2 has markedly the slowest fluid flow. It is important to contrast these results for the fluid flow with results for the local absolute strain rate close to the surface (Fig. 5*A* and see *Materials and Methods* for details). Since the shape of the tibia and the region of evaluation were very similar, the resulting curves for the strains at the endocortical and periosteal surfaces are almost identical for the three animals. In all mice, the maximum strain rate was higher at the periosteal surface by about 35% compared to the endocortical surface.

## Discussion

In this study, we first structurally described the architecture of the LCN in mice tibiae after 2 wk of mechanical loading. Then the network information was used for a functional interpretation by calculating the fluid flow through the LCN, predicting local strains in the bone using FE analysis, and measuring the bone’s mechanoresponse (sites of formed, resorbed, and quiescent bone). Compared to human osteonal bone (36), the LCN of mice is more than three times denser with an average value of the canalicular density of 0.27 µm/µm^3^. Noteworthy is the strong spatial heterogeneity of the network (47–49) with an intracortical band of loose network and high porosity within the tibial cross-section. This band correlates spatially with the woven bone found in murine bone and islands of calcified cartilage, which are thought to be a remnant of early life (47, 50). This heterogeneity implies a caveat when reporting changes in the network architecture of mice due to disease or treatment. Only a 3D mapping of large bone volumes yields reliable values for parameters characterizing the LCN architecture.

The key message of this study is that the prediction of bone’s mechanoresponse is improved by considering the architecture of the LCN compared to relying on the local mechanical strain only. The network architecture crucially influences the fluid flow through the LCN and, consequently, the mechanical stimulation of the osteocytes. Consideration of the LCN architecture leads to qualitatively different results than taking into account strain alone. We found that fluid flow through the LCN allows one to predict the sites of bone formation correctly in individual animals and on different bone surfaces (endocortical vs. periosteal). Independent of the predictor, the model acts on a common length scale, which is defined by the size of a typical finite element (>10 µm). With the number of lacunae (∼2,300) similar to the number of FEs (∼2,750), this common length scale allows an alternative comparison between the two predictors calculating the lacunar pressure in two different ways (*SI Appendix*, Fig. S5 and *Supplementary material*): The lacunar pressure considering the LCN is compared with the case of closed-off lacunae, in which the lacunar pressure corresponds to the strain for a compressible fluid. Moreover, our analysis allows to identify mechanisms of how the local network architecture modulates the velocity of the local fluid flow. A mechanism to locally reduce the fluid flow is the structural incorporation of vascular channels while the bone is laid down (Fig. 1*B*). The fluid flow patterns (Fig. 3*A*) demonstrate how vascular channels can act as additional sinks/sources, and thereby shield the nearby bone surface from fluid flow. Although this shielding effect of vascular canals was already hypothesized based on continuous FE modeling (51), our data confirm how the exact position of the vascular canals and the interplay with the LCN architecture affect bone (re)modeling. The interpretation of fluid flow through networks is based on the principle that fluid flows predominantly through the path of least resistance among a set of alternative paths within networks. Since vascular channels are located especially near regions with a less dense and less connected LCN (and, therefore, of high flow resistance) (Fig. 1*B*), the path toward these vascular channels is the preferred flow path, thereby reinforcing their “shielding effect.” A very different mechanism is responsible for the high flow velocities close to surfaces exhibiting a strong mechanoresponse. Here, when approaching the bone surface, the network converges, with the fluid flow being “funneled” into fewer canaliculi (Fig. 1*C*). The (practically) incompressibility of the fluid causes an acceleration of the fluid, once a reduced number of canaliculi are available.

A topic that has been largely neglected by bone researches, because it is so hard to address, is the problem of signal integration: How are the biological signals, which are produced by various osteocytes as a response to fluid flow, then added up and transported to the surface of the bone to orchestrate the behavior of osteoblasts and osteoclasts after mechanotransduction? In the scope of our model, we therefore asked, how should the averaging over the fluid flow velocities in the LCN be performed to obtain a good predictor for bone’s mechanoresponse? Our analysis allows us also to speculate on this point. In our fluid flow analysis, the predictive power of the bone’s mechanoresponse is best, when the weighted average of the flow velocity (*Materials and Methods*) is restricted to canaliculi only tens of micrometers away from the bone surface (i.e., for the evaluation of Fig. 5 *A* and *C*, *R* = 15 µm was chosen in Eq. **1**; *Materials and Methods*). If the mechanosensitivity is largely restricted to network contributions close to the surface, this would have important implications for bone adaptation. The continuous bone apposition at the periosteal surface in mice and humans (52) could be used for a continuous adaptation of the network architecture to modulate the flow through it. A feedback mechanism has been hypothesized based on the experimental finding that the osteocyte density correlates with bone apposition rate (53, 54). Also, the strong heterogeneity in LCN architecture in mice can be associated with differences in bone formation rate (55). For example, this could explain why some surfaces have a more sensitive “funneling LCN architecture” (Fig. 1*C*).

The intricacy of the LCN architecture makes model assumptions necessary. Even with the restriction of the imaging depth to roughly 50 µm, the imaged volume of the mouse tibia contains ∼4.5 million canaliculi. The 3D network architecture of the canaliculi in this 50-µm-thick volume is illustrated in 3D rendered videos (see Network_animation_mouse1.avi in ref. 46). Standard confocal microscopy cannot resolve the diameter of the canaliculi. Consequently, the annulus region, in which fluid can flow, was assumed to have a cross-sectional area of 0.045 µm^2^ for all canaliculi. The fluid does not flow freely through this annular space but is substantially impeded by its fibrous filling. As a consequence, the dependency of the fluid flow velocity on the unknown dimensions of the annular space is limited (12). *SI Appendix*, *Supplementary material* includes additional simulation results demonstrating the robustness of our fluid flow predictions with respect to a random variability of canalicular permeabilities. The large number of canaliculi also restricts the accuracy of the fluid flow calculations compared to previous works, which analyzed the fluid flow through single lacunae with their adjacent canaliculi (31, 56) or within single canaliculi (57, 58). In particular, our model does not consider any interplay between the fluid flow and the shape of osteocyte bodies. Not only these fluid simulations, but also microfinite-element calculations (59) and experimental strain measurements (60) show local heterogeneities in the flow and strain, respectively, due to specifics of the lacunar and canalicular shape. On these submicrometer length scales future modeling approaches have to incorporate relevant ultrastructural information on the organization of bone lamellae and mineralized collagen fibers (61).

The fluid flow is also predicted based on the LCN architecture before the mechanoresponse of new bone formation and resorption. In reality, the adaptation process is more dynamic, so that the first bone (re)modeling would already have an influence on the fluid flow pattern. Additionally, osteocytes may be even able to actively manipulate the permeability of certain canaliculi, for example by perilacunar/canalicular remodeling (62) and/or obstructing the fluid flow with their cell processes. Such an active control of the fluid flow would allow indirect communication between osteocytes (63). Our approach can be justified by the assumption that there is a time delay between mechanosensing and actuation in terms of bone formation and resorption. Consequently, to predict bone’s mechanoresponse, one should analyze the mechanical stimulation in its recent past. The time-consuming analysis was limited to three mouse tibiae only. Based on our results, we suggest designing further studies including larger sample cohorts to enable a thorough statistical analysis. Efficient progress in our understanding of bone’s mechanobiology could also be made by extending the analysis to the trabecular bone compartment (26), different bones in the mouse, larger bone volumes (64), and different small animals. The strength of our model approach is that an assessment of fluid flow in the whole network can be performed, although the large number of canaliculi poses a challenge to computational resources available nowadays. The result of the analysis are patterns of fluid flow velocity with a striking spatial heterogeneity (Fig. 3*A*), especially when compared to the smooth strain patterns (Fig. 3*B*).

While fluid flow is an excellent predictor of bone formation, this holds much less for resorption. A valid prediction is that no resorption was found when the average fluid flow velocity at the surface is above 5 µm/s. However, the resorption at the periosteal surfaces was noticeably less compared to the endocortical surfaces, despite the surfaces having very similar fluid velocities. We want to provide four possible reasons for shortcomings of model predictions: 1) Especially for the case of resorption, it has been proposed that microdamage in the bone could act to trigger the process (65–67); 2) since similar endocortical bone resorption is also observed in the nonloaded limb (42), this could be a response uncoupled to mechanics and related to shape changes of the whole tibia (64). A future fluid flow analysis as proposed in this study on the nonloaded limb is important to confirm that the obtained findings can be extended to bones under physiological loading. 3) To understand details of mechanotransduction, a more microscopic viewpoint than taken here is necessary, to consider the role of integrins (25) and the glycocalyx (14, 68–70). 4) Although this study focuses on biomechanical aspects of bone adaptation, we do not want to give the wrong impression that molecular and cell biological aspects should take a back seat. In the end, cells must be available and they have to comprehend and execute instructions that are provided by mechanical stimulation. Metaphorically speaking, our point of view for bone’s mechanosensitivity is that the LCN is the hardware, on which the biological software can play.

Future work using the approach of the present study should corroborate the potential of applying the fluid flow hypothesis to predict the mechanoresponse of bone. A straightforward extension is to analyze different anatomical locations, mouse strains, mouse ages, diseases that lead to deterioration of the LCN, or other small rodents. A fascinating question is how much the LCN architecture can explain differences in the mechanoresponse of different bones in the human skeleton. For example, our skull does not get resorbed despite a low mechanical loading (71). Also, it is known that in general the LCN architecture changes with age (40). It should be tested whether these changes can be responsible for the decrease in mechanoresponse with age (27, 42, 72). More challenging will be to determine the influence of LCN architecture and fluid flow on bone development and growth. The difficulty is to untie the feedback loops that couple mechanical stimulation, bone growth, and the constant addition of new canalicular network. Finally, an evolutionary perspective on the LCN raises some fundamental questions. The existence of fish, which through evolutionary selection have neither osteocytes nor a LCN (73), suggest that mechanosensation was not the primary function of the osteocytes. Since it seems not a futile undertaking to image the LCN in fossil bones (35), these ancient bone samples could tell us the exciting story of how a new function was introduced into our bones.

## Materials and Methods

### In Vivo Mechanoresponse Experiment and 3D Dynamic In Vivo Morphometry.

Two weeks of controlled loading was applied on the left tibia of three skeletally mature (26-wk-old) female C57BL/J6 mice (The Jackson Laboratory) to provoke a bone (re)modeling response (43). The loading protocol (74) and time-lapse imaging method (75) have been previously reported in detail and are only briefly described here. The loading protocol consisted of 216 cycles/d, 5 d/week, with a 4-Hz triangular waveform. A load of −11 N was applied to induce a peak strain of +1,200 µε, based on previous in vivo strain gauging (74). In vivo µCT scans with a voxel size of 10.5 µm were taken of the mid diaphysis, covering 5% of the tibia length on day 0 and 15 (VivaCT 40; Scanco). Animal experiments were carried out according to the policies and procedures approved by the local legal representative (LAGeSo Berlin, G0168/13). µCT images of the same bone, acquired at different time points, were geometrically aligned in a common coordinate system using a 3D rigid registration algorithm with normalized mutual information as the optimization criterion. The voxels in the fused dataset can then be classified as newly formed, resorbed, or quiescent bone (Fig. 3*C*). The local mechanical strains induced within the bone during the controlled loading experiment were determined using animal specific FE models (Abaqus, Dassault Systemes Simulia). FE models were developed using ex vivo μCT scan (Skyscan 1172; Bruker; 9.91-μm isotropic voxel size) of full tibiae. Material properties were assumed linear elastic, but spatially heterogeneous based on the linear attenuation coefficients extracted from the ex vivo scans and validated by synchrotron computed tomography measurements (see ref. 44 for model details).

### Sample Preparation, Confocal Laser-Scanning Microscopy, and LCN Analysis.

**Fig. 6.**
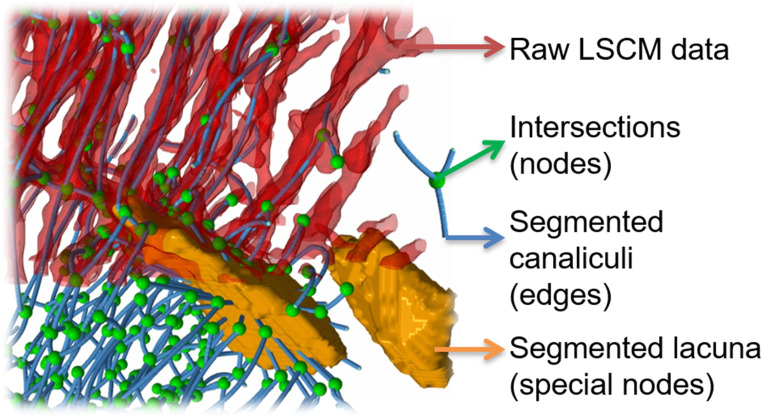
Different stages of the image analysis methods of confocal laser-scanning microscopy (CLSM) data. After thresholding of CLSM raw data with a fixed threshold, segmentation (based on bulkiness and local change in curvature by “expanding” the lacunae into the canaliculi) allows a separation between canaliculi (red tubes) and lacunae (orange blobs) (36). Skeletonization converts the image into a mathematical network with edges representing canaliculi (blue lines) and nodes representing intersection between canaliculi (green spheres) and lacunae.

Confocal laser-scanning microscopy (CLSM) was used after rhodamine staining to image the LCN in whole cross-sections of the mouse tibiae. Tibia samples with only their knee and ankle joints removed were immersed in an ethanol solution with rhodamine 6G for 24 h. This was repeated three times with a fresh rhodamine 6G solution and then embedded in poly(methyl methacrylate) (PMMA) using our previously established protocol (76). The bone was kept wet with ethanol until the PMMA embedding process was finished to prevent crack formation. Based on high-resolution ex vivo µCT scans (5-µm voxel size; SCANCO µCT 50; SCANCO Medical) to define the region of interest, the embedded bones were cut transversally using a diamond wire saw equipped with a 50-µm-thick wire and a stereo microscope (DWS.100; Diamond WireTec) and polished. With rhodamine (molecular size, <2 nm) penetrating all accessible porosities in the sample and attaching at surfaces, the LCN and vascular channels can be imaged in 3D using CLSM (Leica SP8) (6, 47). An image resolution of 370 nm was obtained with a 40× oil immersion lens (Leica, HCX PL APO 40× NA 1.25 oil). Since under these imaging conditions the field of view (0.4 mm) is smaller than the size of a tibial cross-section (about 1.2 mm), 16 image stacks covering the whole cross-section were taken and then stitched using the ImageJ software tool BigStitcher (77). This tool allowed an accurate alignment of canaliculi to ensure their continuity between different image stacks. With an updated version of our custom-made Python software Tool for Image and Network Analysis (TINA) (available in ref. 78), the image dataset was automatically segmented into canaliculi, lacunae, and vascular channels and then converted into a mathematical network consisting of edges (representing canaliculi) and nodes (representing all intersections between canaliculi, including lacunae and vascular channels) (Fig. 6). This network was further analyzed using NetworkX 1.7 (79). The canalicular density (Ca.Dn) was evaluated for (8 µm)^3^ big subvolumes as total length of canaliculi per unit volume. The pore density for these subvolumes was calculated as volume of both lacunae and vascular channels per unit volume.

### Simulation of the Load-Induced Fluid Flow through the LCN.

Circuit theory based on Kirchhoff’s first law was used to calculate the fluid flow velocity in each canaliculus of the network (for details of the model and parameter values used, see ref. 41). Describing the topology of the network by the directed edge-node incidence matrix $A_{ji}$, with elements equal to 1 (or −1) if edge j points toward (or away from) node i, and otherwise 0, conservation of fluid in the network (Kirchhoff’s first law) can be written as $\sum_{j}A_{ji}q_{j}=f_{i}$ with $q_{j}$, the volumetric flow rate through the edge j, and $f_{i}$, the source/sink contribution to the flow of node *i*. Exploiting the definition of the incidence matrix leads to $\Delta p_{j}=\sum_{i}A_{ji}p_{i}$, where $\Delta p_{j}$ denotes the pressure difference over edge j, and $p_{i}$, the pressure at node i. Darcy’s law relates the pressure difference and volumetric flow rate within each edge, $q_{j}=C_{jj}\Delta p_{j}$. The entries in the conductivity matrix $C_{jj}$ can be estimated following the approach of Weinbaum et al. (12), which takes into account that the fluid can only flow in the annulus between osteocyte process and canaliculus wall and that this space is filled with a fibrous matrix. The hypothesis of load-induced fluid flow implies that each node under compression/tension is a source/sink of fluid, respectively. The rate of fluid volume contributed by node i, i.e., the value of $f_{i}$, is calculated as the product between two quantities: 1) the volume of the porosity corresponding to node i, which equals half of the volume of all of the canaliculi connected to the node plus the volume of the lacuna in case that the node represents a lacuna; 2) the local mechanical volumetric strain rate, which can be estimated from the tibia loading protocol in combination with FE calculations. Combination of Kirchhoff’s first law, Darcy’s law together with the values for $f_{i}$, the volumetric flow rate $q_{j}$ in each edge j can be calculated, which can be easily converted into an average velocity in the canaliculus j, $v_{j}$, since $q_{j}=v_{j}A$ with A, the cross-sectional area of the annulus region between cell process and canaliculus wall. This average velocity is linearly related to the shear force on the cell membrane of osteocytes (12). The final step remaining is “to integrate” this information about fluid flow velocities to obtain a predictor for the mechanoresponse at the bone surfaces. First, both the endocortical and periosteal surface are discretized in 180 arc-shaped elements each covering an angle of 2°. For each element, a weighted mean of the fluid flow velocity in all canaliculi, which are located in the “wedge” of 2° opening angle, was calculated. The weighting follows the idea introduced by Mullender and Huiskes (80) that contributions closer to the surface are more important than farther away from it. Using an exponential weighting, the quantity that should predict the mechanoresponse of loaded bone in an element of the endocortical or periosteal surface was defined as follows:

$${\left(\sum_{j}l_{j}\right)}^{-1}\sum_{j}v_{j}l_{j}\exp\left[\left(\frac{r_{j}}{R}\right)\right],$$ [1]

with $l_{j}$, the length of the canaliculus j, and $r_{j}$, the distance from the bone surface to canaliculus j; weighted averaging is executed over all canaliculi within the wedge of 2° opening angle. For the presentation of the results, a 30° triangular moving average was used to smoothen the angular dependence. Since the value of R is unknown, a parameter study was performed with the result that the mechanoresponse could be well predicted for 3 < *R* < 30 μm with an average RMSE below 12 µm. For the results of Fig. 4, the choice was *R* = 15 μm.

## Supplementary Material

Supplementary File

## Data Availability

Raw microscopy data have been deposited in the Open Access Data Repository of the Max Planck Society (https://edmond.mpdl.mpg.de/imeji/collection/0fK7DWn6fkD13hs). All study data are included in the article and *SI Appendix*.
