# Supplementary material for: The mechanoresponse of bone is closely related to the osteocyte lacunocanalicular network architecture
Source: Proc Natl Acad Sci U S A. 2020 Dec 7;117(51):32251–9. doi: 10.1073/pnas.2011504117 (PMC7768754; doi:10.1073/pnas.2011504117)
Supplement: Supplementary File [file pnas.2011504117.sapp.pdf]

Variation between animals

Figure S1 shows the variation in lacunocanalicular network (LCN) properties between the three mice. The starting point of the analysis are images of the LCN obtained with confocal laser scanning microscopy (CLSM) after rhodamine staining (Fig. S1A). The map of the spatial distributions of canalicular density, Ca.Dn, (i.e., total length of canaliculi per unit volume) (Fig. S1B) reveals a ring-like structure of low network density, which runs eccentrically in the cortex in all three mice. Although the overall location of the low network density ring is similar in each mouse, individual differences can be observed (exact location of the ring, width of the ring, etc.). Evaluation of the pore volume fraction (i.e., volume of both lacunae and vascular canals per unit volume) (Fig. S1C) demonstrate that regions of high porosity spatially correlate with regions of low network density in all three mice (Fig. S1B, C). In mouse 3 vascular canals running more or less horizontally between the endocortical and the periosteal surface can be observed.

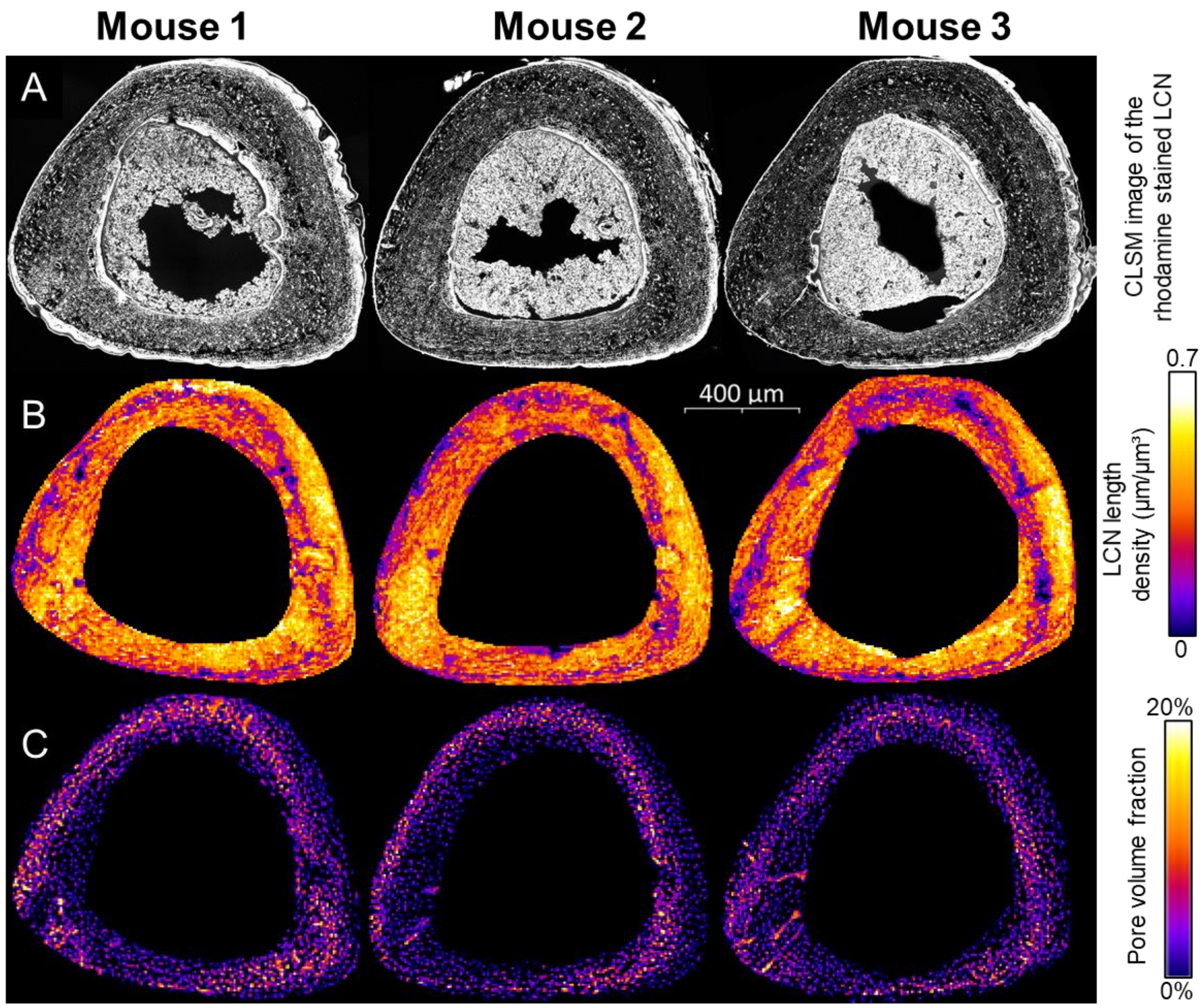

Fig. S1. A) Confocal laser scanning microscopy (CLSM) images covering the complete cross-section of the tibia after rhodamine staining. For reasons of presentation, a single 2D section of the 3D image stacks is shown. B) Maps of canalicular densities Ca.Dn. (i.e., total length of canaliculi per unit volume) C) Maps of pore volume fractions (i.e., volume of both lacunae and vascular canals per unit volume). The maps in B) and C) are obtained after averaging over the imaging depth of 50 µm.

The remodeling of the three mice in terms of newly formed and resorbed bone after two weeks of controlled loading is shown in Figure S2A. The found patterns are similar between the three mice with strongest bone formation at the posterior endocortical and periosteal surfaces. The spatial strain maps (Fig. S2B) for the three mice closely resemble each other with the regions of tensile and compressive strain caused by the bending of the tibia. The resulting fluid flow patterns (Fig. S2C) have a "hybrid" character combining features of the strain maps and features determined by the architecture of the lacunocanalicular network. Again, while the overall pattern of the fluid flow velocity is similar in all three mice, it is not difficult on closer inspection to find individual differences in the spatial heterogeneity.

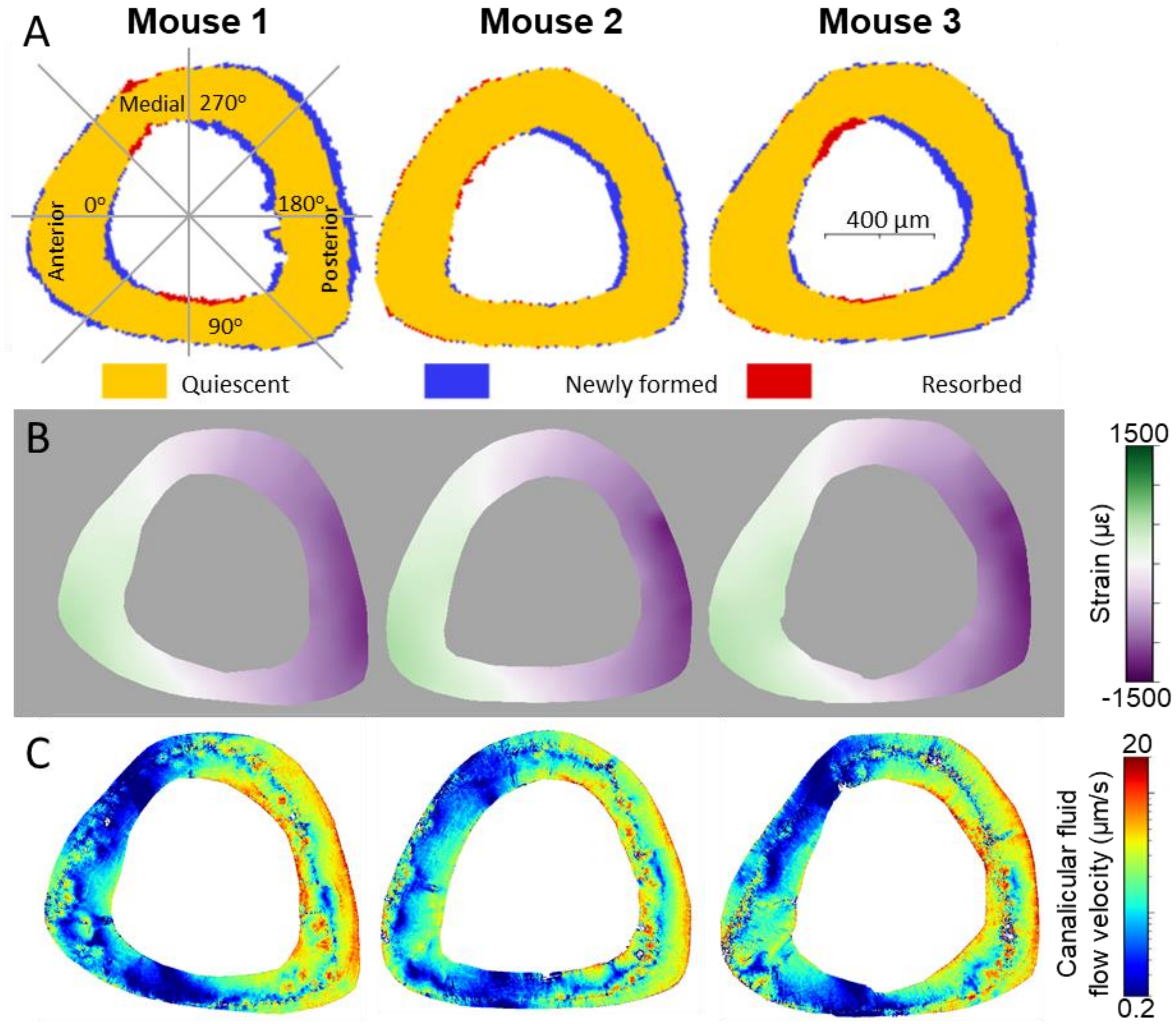

Fig. S2. A) Outcome of the in vivo μCT experiment showing where in the diaphyseal region of the tibiae, bone was formed or resorbed in response to controlled dynamic mechanical loading over two weeks (blue denotes newly formed bone, red resorbed bone, and yellow quiescent bone; 2D cross-section of an imaged 3D volume). B) Spatial distributions of the strains induced by the maximum load during the in vivo loading experiment, calculated using finite element modeling. Green colors correspond to tensile, violet to compressive strains. C) Patterns of fluid flow velocities through the lacunocanalicular network (LCN). Based on the loading conditions from B) and the 3D network architecture, the fluid flow velocity is calculated in each canaliculus using circuit theory. The fluid flow velocity information of all the canaliculi was rendered in a 3D image stack. For reasons of presentation this 3D image is averaged over the imaging depth to obtain the shown flow pattern.

Robustness of fluid flow results – effect of variation in canalicular permeability

The circuit model of the fluid flow through the canaliculi assumes that all the canaliculi in the network have the same permeability and that the annular region between cell process and canalicular wall is always the same (0.045 µm^2). This assumption had to be made since ultrastructural information of the cell process/canaliculus is not available for the large bone volumes of our study (i.e., whole tibial cross-section). To test the robustness of this assumption of uniform canalicular permeability, we introduced a random variability in this model parameter. Values were informed by electron microscopy studies of the canalicular ultrastructure in humeri of BalB/c mice (1). The large variability (diameter of the canaliculi 259 ± 129 nm; diameter of the cell process 104 ± 69 nm) was attributed – among others - to a change of diameter along the canaliculus with large values close to the cell body.

Our protocol of testing the robustness of our fluid flow predictions was: (i) assigning to each canaliculus a value for the canalicular permeability, chosen randomly from a Gaussian distribution. Specifically, in the model the conductivity of each canaliculus j was redefined as $C_{var,jj} = X_j \cdot C_{jj}$, where the variable X_j was sampled from a Gaussian distribution with $\mu = 1$ and $\sigma = 0.25$; (ii) running the simulation and predicting the site and amount of bone formation/resorption based on the fluid flow as usually (i.e., as described in the Materials and Methods section); (iii) repeating the random assignment and the evaluation of the fluid flow for 10 independent runs per tibia; (iv) evaluating the robustness by using the root-mean-square-error (RMSE) to compare predicted and measured mechanoresponse.

Figure S3 shows representative results for a single simulation. The frequency histogram of the relative change between the simulation without variability of the canalicular permeability compared to with variability, showed a slightly asymmetrical distribution with a sharp peak at 0% change (Fig. S3A). The result that the fluid flow is hardly affected in most of the canaliculi is also reflected by the accumulation of points on the diagonal $x = y$ (Fig. S3B), when plotting the fluid flow velocity in each canaliculus without variability vs with variability. The change of the fluid flow velocity averaged over all canaliculi was around +18% for all three mice, which was mostly caused by a fraction of all canaliculi, in which the fluid flow velocity more than doubled.

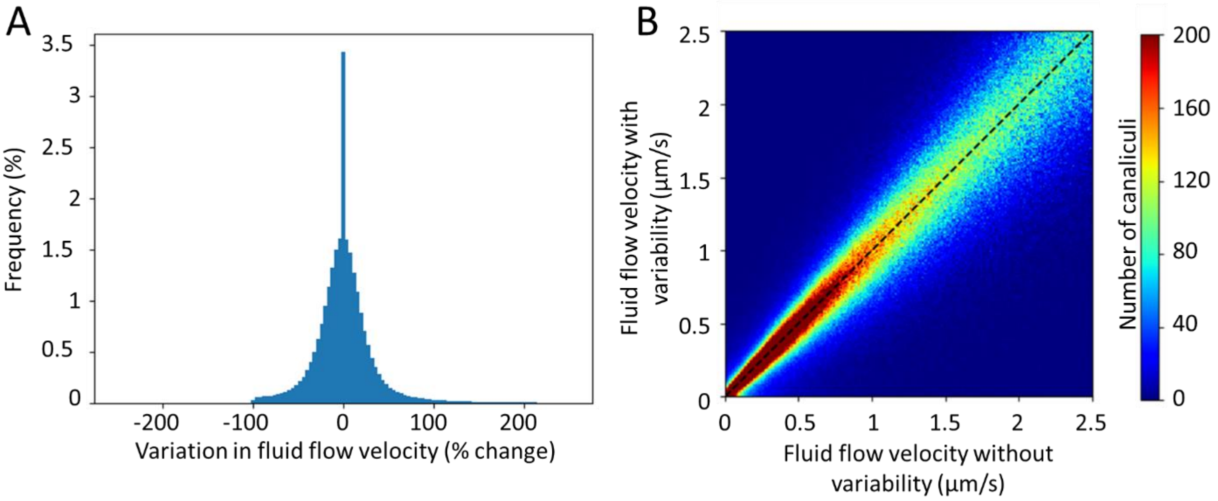

Fig. S3. A) Histogram of one simulation to demonstrate the relative changes of fluid flow velocity as a result of a variation of the canalicular permeability. The permeability was changed with a factor taken from the normal distribution with μ = 1 and σ = 0.25. B) Scatter plot showing the relationship between simulated fluid flow velocity with variation in canalicular permeability and the fluid flow

velocity without variation in permeability. A dashed line with a slope of 1 has been added to illustrate the line of 0% change in fluid flow velocity.

Figure S4 shows the predictions of bone remodeling based on a model with a variability in canalicular permeability. The green curves are the result presented in Figure 5C (solid line: periosteal; dotted line: endocortical) and serve as reference for the case without variability. Two results can be observed: Firstly, there is hardly any variability between the outcome of the 10 independent runs, which makes it even impracticable to show error bars. Secondly, the outcome is clearly distinct from the reference simulations with constant canalicular permeability with a clear trend that on average fluid velocities are increased. We interpret this increase in fluid velocities as the consequence of very small values of the permeability being randomly assigned to some of the canaliculi. With canaliculi being practically sealed off, the fluid flow has to take detours, which lengthens the flow path to the free surface. A longer flow path results in an increase of the average fluid flow velocity, an effect that we observed also on our study of osteon-in-osteons (2).

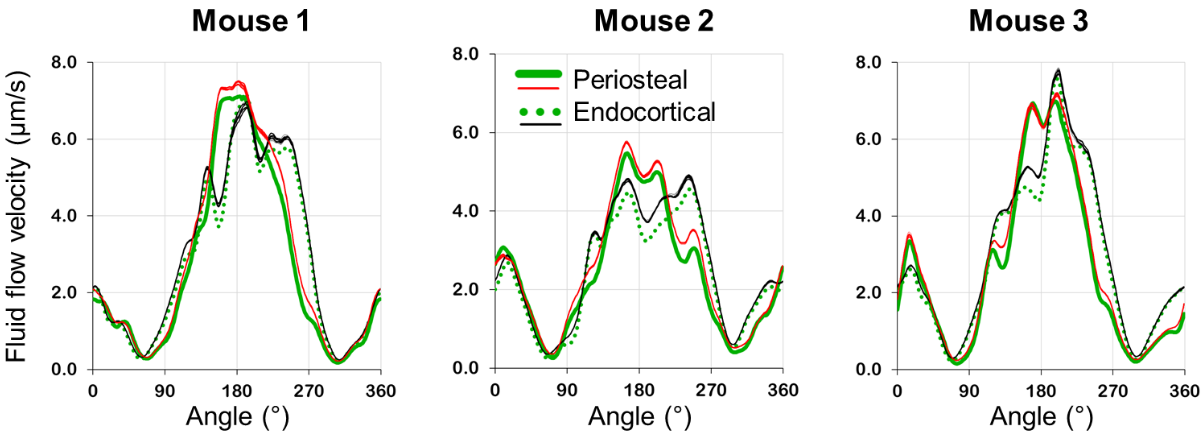

Fig. S4. The effect of variation of the canalicular permeability on the fluid flow velocity predicting bone formation/resorption. Thick green lines (solid: periosteal; dotted: endocortical) show the fluid flow velocity of the model without variation in canalicular permeability. Thin lines are 10 simulations where canalicular permeability was randomly varied (red: periosteal; black: endocortical). These lines are so close together that only at specific positions a separation between them can be observed.

The quantitative assessment of the prediction quality in terms of RMSE yielded the following values:

RMSE ± Standard deviation of RMSE [µm]	Mouse1	Mouse2	Mouse3
Flow without variability - endocortical	21.8	13.7	12.8
Flow with variability – endocortical	21.3 ± 0.02	14.0 ± 0.04	14.6 ± 0.02
Flow without variability - periosteal	16.7	9.0	13.0
Flow with variability - periosteal	13.1 ± 0.02	8.5 ± 0.04	10.8 ± 0.02

From these results, we conclude that even a substantial variation in the canalicular permeability hardly changes the predictions based on the fluid flow – the variation even slightly improved the prediction quality. However, this demonstration of the robustness of the model predictions is valid only for a random variability of the canalicular permeability. It cannot be simple extended, for example, to the case that ultrastructural features adapt to the fluid flow and lead to a correlation between flow velocity and canalicular permeability.

Direct comparison of predictions based on lacunar pressure

For a fair comparison between a prediction of the mechanoresponse considering only strain, or considering strain plus the architecture of the lacunocanalicular network, the model has to act on a common length scale. Independent of the predictor, the starting point of the model is the local strain in the tibia (i.e., strain acts as the “prime mover”), i.e. the common length scale in the model is defined by the size of a typical finite element (> 10 µm). Although we imaged the LCN with a spatial resolution of 370 nm, the flow through the LCN was modeled using a so-called lumped-element approach at a comparable length scale, e.g. the distance between lacunae, which is several micrometers. This common length scale is underlined by the fact that the number of finite elements (~ 2750) is similar to the number of osteocyte lacunae (~ 2300) in our volumes of interest.

A quantity that can be evaluated on this length scale and that can be used for comparison of the two predictions is the lacunar pressure. Evaluating the lacunar pressure under two different boundary conditions provides an interesting view on a mechanoresponse prediction based on strains only, and considering in addition the LCN architecture. In the first scenario, we assume that the lacuna is closed off (i.e., the fluid cannot be drained by canaliculi). For a compressible fluid, the pressure in lacuna due to loading is directly proportional to the local strain. In the second scenario, we take into account the LCN, i.e., under loading the fluid can now escape via the canaliculi emerging from the lacuna resulting in a pressure release. In this case, the pressure in the lacuna reflects the architecture of the LCN and comprises the same information than the fluid flow velocity in the canaliculi (analogous to either voltage or current carrying the same information in an electrical circuit). Figure S5 shows now that the external loading leads to different pressures in the lacunae depending whether the lacunae are closed off or the lacunae are connected by canaliculi. Deviations from a linear relationship between the two quantities can be interpreted as the action of the LCN architecture on the fluid flow through the network.

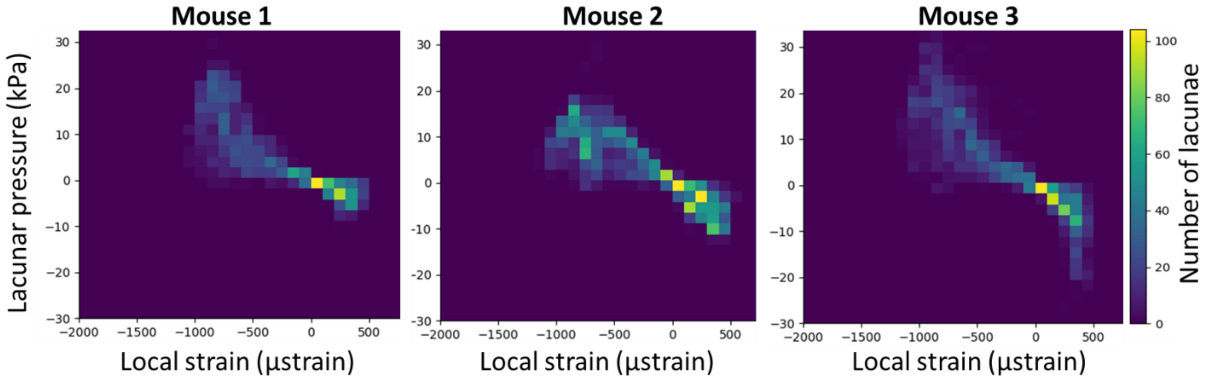

Fig. S5. Lacunar pressure when considering that under dynamic loading fluid is drained via the canaliculi (y-axis) as function of strain (i.e., the pressure in the lacuna for a compressible fluid, when the lacuna is assumed to be closed off) (x-axis). The color code shows the number of lacunae with a specific pressure-strain-relationship. Deviations from a linear relationship between both quantities show the impact of the network architecture.

Videos:

Four videos are deposited in the Open Access Data Repository of the Max Planck Society (Edmond) and can be accessed through the following address:
https://edmond.mpdl.mpg.de/imeji/collection/0fK7DWn6fkD13hs

The aim of the videos is to convey two messages: 1) the description is based on a three-dimensional network although the plots typically show two-dimensional projections of the evaluated quantity and 2) how the transition occurs from the discrete network to the "continuous plots" shown, for example, in Figure 3C of the spatial distribution of the fluid flow.
Three videos [clsm_mouse1/2/3.avi] show the succession of images in the image stack of the whole tibia over the image depth of 65 µm. The last video [Network_animation_mouse1.avi] starts with the discrete network structure and shows the transition to the continuous plots of the fluid flow velocity.

References:

1. L. D. You, S. Weinbaum, S. C. Cowin, M. B. Schaffler, Ultrastructure of the osteocyte process and its pericellular matrix. *Anat Rec Part A* **278a**, 505-513 (2004).
2. A. F. van Tol *et al.*, Network architecture strongly influences the fluid flow pattern through the lacunocanalicular network in human osteons. *Biomech Model Mechan* **19**, 823-840 (2020).
